# Complete Genome Sequence of Serogroup B Neisseria meningitidis Strain C311

**DOI:** 10.1128/MRA.00788-21

**Published:** 2021-10-21

**Authors:** Freda E.-C. Jen, John M. Atack, Yuan Zhang, Jennifer L. Edwards, Michael P. Jennings

**Affiliations:** a Institute for Glycomics, Griffith University, Brisbane, Queensland, Australia; b Center for Microbial Pathogenesis, The Abigail Wexner Research Institute at Nationwide Children’s Hospital, Columbus, Ohio, USA; c Department of Pediatrics, The Ohio State University, Columbus, Ohio, USA; University of Maryland School of Medicine

## Abstract

Neisseria meningitidis strain C311 has been widely used to study meningococcal pathogenesis in the past 30 years, but its genome is not available. Here, we report that the complete C311 genome is 2,311,508 bp in length, contains a total of 2,274 genes, and has a GC content of 51.25%.

## ANNOUNCEMENT

Neisseria meningitidis is a Gram-negative bacterium that can cause acute bacterial meningitidis and life-threatening sepsis. N. meningitidis serogroup B strain C311 was originally isolated from a patient in Alder Hey Children's Hospital (Liverpool, England) in the 1980s (1). It was first used to study the virulence factor pili (type IV fimbriae) (1, 2). Subsequently, C311 has been adopted as a model strain for meningococcus-host cell interactions, largely driven by a series of studies by Mumtaz Virji and colleagues, including pilus characterization and analysis of the role of pili, opacity proteins, lipooligosaccharides, and capsule in mediating host cell interactions (3–15). Nearly 100 publications have used N. meningitidis strain C311 to study pathogenesis in the past 30 years, but a complete genome of C311 was not available. N. meningitidis undergoes random switching of gene expression by phase variation (16, 17) and antigenic variation (18) for many important surface virulence factors, which contributes to immune evasion and host adaptation during host infection. N. meningitidis strains contain ∼65 to 100 potentially phase-variable genes (16, 17). To study N. meningitidis pathogenesis, long-read whole-genome sequencing (WGS) (single-molecule real-time [SMRT] sequencing) is the best tool for obtaining closed complete genomes. Here, SMRT sequencing was used to determine the genome sequence of N. meningitidis strain C311. We report its closed, annotated whole-genome sequence, and this will be a useful tool for studying phase-variable and antigenic variation genes in N. meningitidis and meningococcal pathogenesis.

N. meningitidis strain C311 was obtained from the Department of Paediatrics, Oxford University, in 1996 and was provided and maintained at −80°C as a frozen stock in brain heart infusion (BHI) broth (Oxoid Limited, Ireland) with 40% glycerol, with no subculture. A sample scraped from the top of the frozen stock was grown overnight on BHI agar (Oxoid) at 37°C in 5% CO_2_ and was subcultured heavily onto a fresh BHI agar plate and grown for an additional 4 h. Cells were harvested, genomic DNA was isolated using the GenElute kit (Sigma-Aldrich), and PacBio long-read sequencing was carried out at SNPsaurus (Eugene, OR). The sequencing library was prepared using the SMRTbell Express template preparation kit v2.0 (Pacific Biosciences, Menlo Park, CA) according to the manufacturer’s protocol. Samples were pooled into a single multiplexed library, and automatic DNA size selection was performed with the BluePippin system (Sage Science, Beverly, MA) with a 0.75% DF Marker S1 high-pass 6- to 10-kb v3 cassette (Sage Science) according to the manufacturer’s recommendations. A size-selection cutoff value of 8,000 bp (BP start value) was used. The size-selected SMRTbell library was annealed and bound according to the SMRT Link setup (Pacific Biosciences) and was sequenced on a Sequel II system (v1.0 chemistry) at SNPsaurus. Raw reads were converted to the fasta format with SAMtools (19). Flye v2.8 (20) with default parameters was used to assemble and polish the C311 genome. The assembly quality was assessed using BUSCO v3 (21) with default parameters. An average coverage of 243-fold was obtained. The assembled sequence was annotated using PGAP v5.2 during NCBI GenBank submission of the closed genome sequence. Information on the sequenced N. meningitidis C311 strain is summarized in Table 1.

**TABLE 1:** Summary of information for the closed annotated genome sequence for N. meningitidis strain C311

Parameter	Finding
Strain	C311
Isolation source	Cerebrospinal fluid
Genome size (bp)	2,311,508
Genome coverage	243
Total no. of reads (bp)	596,891,955
Mean read length (bp)	19,211
GC content (%)	51.25
No. of genes	2,274
No. of coding sequences	2,199
GenBank accession no.	CP079941
SRA accession no.	SRX11501812

### Data availability.

The closed annotated genome and WGS reads have been deposited in the NCBI database. The accession numbers for the closed genome and the raw data are provided in Table 1. The master record for the WGS reads and closed annotated genome can be found in the NCBI database under BioProject accession number PRJNA748166.
